# Construction of a predictive model for cervical lymph node metastasis in papillary thyroid carcinoma

**DOI:** 10.3389/fonc.2025.1549148

**Published:** 2025-05-15

**Authors:** YanHong Hao, Yuan Su, Yanan Li, Qiaohong Pan, Liping Liu

**Affiliations:** ^1^ Department of Ultrasound, First Hospital of Shanxi Medical University, Taiyuan, China; ^2^ Department of Ultrasound, Heping Hospital Affiliated to Changzhi Medical College, Changzhi, China; ^3^ Department of Interventional Ultrasound, First Hospital of Shanxi Medical University, Taiyuan, China

**Keywords:** papillary thyroid cancer, lymph node metastasis, risk factors, predictive model, nomograms

## Abstract

**Background:**

In oncology, the relationships among cervical central lymph node metastasis (CLNM), biochemical tests, and ultrasound characteristics in patients with papillary thyroid cancer (PTC) remain controversial. This association is currently not well supported by evidence, which emphasizes the need for further research. Understanding the connection between CLNM, biochemical testing, and ultrasound features is crucial for clinical practice and public health efforts. Research on this topic is still underway and is now receiving much interest. Our goal was to create and verify a basic cervical lymph node metastasis prediction model.

**Methods:**

In this retrospective cohort study, 685 individuals diagnosed with PTC from First Hospital of Shanxi Medical University (n = 560) and Changzhi Heping Hospital (n = 125) participated in the research from January 2020 to October 2022. Patients were randomly assigned to a training set (n=392), an internal test set (n=168), or an external test set (n=125). Comprehensive clinical information, serological indices, and ultrasonography features were obtained for every participant. LASSO (Least Absolute Shrinkage and Selection Operator) and BSR (Best Subset Regression) to select features for model construction. A logistic regression model with filtered variables was constructed. A nomogram was developed based on six risk factors. Receiver operating characteristic (ROC) curves, decision curve analysis, and calibration curves were used to assess the predictive accuracy, clinical utility, and discriminative ability of the nomogram.

**Results:**

Of the 560 individuals, 54.3% (304/560) did not have lymph node metastases, whereas 45.7% (256/560) did. Age, male, nodule size, multifocal lesions, capsular contact or invasion and ill-defined margins were determined to be risk variables via BSR and multivariate logistic analysis. Nomograms were created using these six risk indicators. The prediction model of CLNM had an AUC of 0.884 (95% CI 0.851, 0.916). Both the internal and the external validation results were highly encouraging. Confirming the model’s stability and applicability in different data environments.

**Conclusion:**

We developed a predictive model and nomogram for CLNM in PTC patients, which demonstrated robust performance. This model can guide surgical planning, potentially reducing complications and improving outcomes.

## Background

PTC is the most prevalent histologic subtype of all thyroid cancers (1, 2). CLNM has an inert clinical course and usually has a good prognosis; however, patients with CLNM face a higher risk of local recurrence and a significant increase in the frequency of distant metastases with advancing N stage, leading to a poorer prognosis (2). Conventional surgery routinely involves central zone lymph node dissection, but unnecessary central zone lymph node dissection can lead to laryngeal recurrent nerve injury, whereas a larger surgical scope of cervical lateral zone lymph nodes may lead to more complications and diminish patient quality of existence (3). Therefore, accurate preoperative prediction of lymph node metastasis is crucial for guiding clinical surgical planning.

The simplest and least expensive method for assessing PTC lymph node metastasis is ultrasound; however, the sensitivity of detection is low because the local anatomical structure influences the appearance and characteristics of centralized lymph nodes, and their preoperative ultrasound is often free of abnormalities (4). Despite its limitations, ultrasound remains a cornerstone in the evaluation of thyroid nodules. Recent studies have explored the potential of combining ultrasound features with clinical and biochemical markers to improve the prediction of CLNM in PTC patients. However, the predictive effectiveness of such multimodal approaches has not been fully elucidated, and there remains a significant gap in the literature regarding the optimal integration of these factors for clinical use.

In this study, we aimed to address this gap by developing a comprehensive clinical prediction model that integrates ultrasound features, clinical parameters, and biochemical markers to provide a practical tool for surgeons to identify patients at risk of CLNM and guide surgical treatment strategies.

## Materials and methods

### Individuals and study design

A total of 685 individuals from two medical institutions from January 2020 to October 2022 were enrolled in this research. Patients from First Hospital of Shanxi Medical University (560) were randomly allocated into a training set (392) and an internal test set (168) at a 7:3 ratio. Patients from Changzhi Heping Hospital (125) were included as an external test set. Patient clinical data, including general clinical characteristics (age, sex), thyroid serological indicators [free thyroxine (FT4), free triiodothyronine (FT3), thyroglobulin (Tg), thyroid stimulating hormone (TSH), antithyroglobulin (TG-Ab), and antithyroid peroxidase (TPOAb)], ultrasound characteristics (tumor size, tumor location, capsule contact, margin, echogenicity, shape, taller-than-wide, composition, intranodal calcification and the number of nodules). The inclusion criteria included: 1) patients who had their first thyroid surgery, with a confirmed diagnosis of PTC by pathology and documented records of cervical lymph node pathology; 2) complete thyroid ultrasound examination within 2 weeks prior to surgery; 3) laboratory examination of thyroid serum markers within 2 weeks prior to surgery; and 4) ultrasound examination to detect the nodule, which was subsequently confirmed by postoperative pathology. The exclusion criteria were 1) incomplete clinical data; 2) unclear ultrasound images or incomplete data that could not be analyzed; 3) the presence of other malignant tumors and 4) postoperative pathological examination revealing the presence of non-PTC components within the lesions, such as follicular adenoma, atypical hyperplasia, medullary carcinoma, anaplastic carcinoma, and metastatic carcinoma. This study adhered to the principles of the Declaration of Helsinki.

### Ultrasound instruments and methods

Ultrasound characteristics of the thyroid nodules were assessed via an Aplio i800 (Canon, Japan) and a Hitachi Arietta 850 System (Hitachi, Japan), which were equipped with probe frequencies of 18 MHz and 5–15 MHz, respectively. The ultrasound characteristics included the following: maximum diameter of the nodule, location, margin, echogenicity, shape, presence of capsular invasion, taller than wide, composition of the nodule, intranodal calcification and number of nodules. All ultrasonographic features were analyzed independently by two ultrasonographers who had been performing ultrasonography for more than 5 years, and in the case of inconsistent results, a third physician discussed and made the diagnosis. To reduce interobserver variability in the interpretation of the ultrasound characteristics of nodules (5). The operators ultrasound skills have been trained through a standardized and unified three-year training program at medical institutions. The ultrasound features of the thyroid nodules were described in accordance with the ACR TI-RADS criteria (6). The kappa coefficient is used to assess the consistency between these two ultrasound physicians. To evaluate the overall consistency, the average κ value is calculated on the basis of the k values assessed for each variable.

### Clinical and biochemical indicators of thyroid function

The patients’ age, sex and thyroid serological indicators, namely, FT3, FT4, TSH, Tg, Tg-Ab, and TPOAb were collected 2 weeks prior to surgery. Values that exceeded normal were considered high, whereas those that did not were considered normal.

### Definitions

The calcification points ≤ 2 mm in microcalcification and >2 mm in calcification are coarse calcifications. Hashimoto’s thyroiditis (HT) was defined on the basis of the patient’s positive preoperative levels of TPOAb and TgAb and the appearance of alterations in the features of the ultrasound images. Capsular contact was classified into three categories: far from the thyroid capsule (≥ 2 mm), contact with the thyroid capsule (< 2 mm) and invasion or breakthrough of the thyroid capsule. The margin is defined as smooth and irregular, with irregularities, including ill-defined and spiculated or microlobulated edges (7).

### Data statistics

Data analyses were performed with Free Statistics version 2.1 (http://www.clinicalscientists.cn/freestatistics/, Beijing, China) and R version 4.2.2 (http://www.R-project.org, The R Foundation). Statistical significance was determined at *P* < 0.05 (two-sided). At a 7:3 ratio, the dataset was divided into a training set and a validation set. Continuous variables that are normally distributed are documented as the means with standard deviations (SDs), and categorical variables are reported as the number and percentage of patients in each category. Independent sample t tests were used for intergroup comparisons; chi-square (χ^2^) tests were used for intergroup comparisons of count data. To address missing data in the analysis, multiple imputation by chained equations (MICE) was utilized.

Multiple stages were executed for the construction and validation of nomograms aimed at predicting CLNM. First, BSR and LASSO regression were applied to select potential risk factors, and the two screening methods were compared. Next, we performed a multivariable logistic regression analysis to pinpoint the independent risk factors for CLNM and then presented the outcomes in a nomogram format. ROC decision curve and calibration analyses were subsequently used to evaluate the model’s discrimination ability and calibration accuracy. Furthermore, the model’s robustness was ensured through external validation via an independent dataset.

## Results

**Figure 1:**
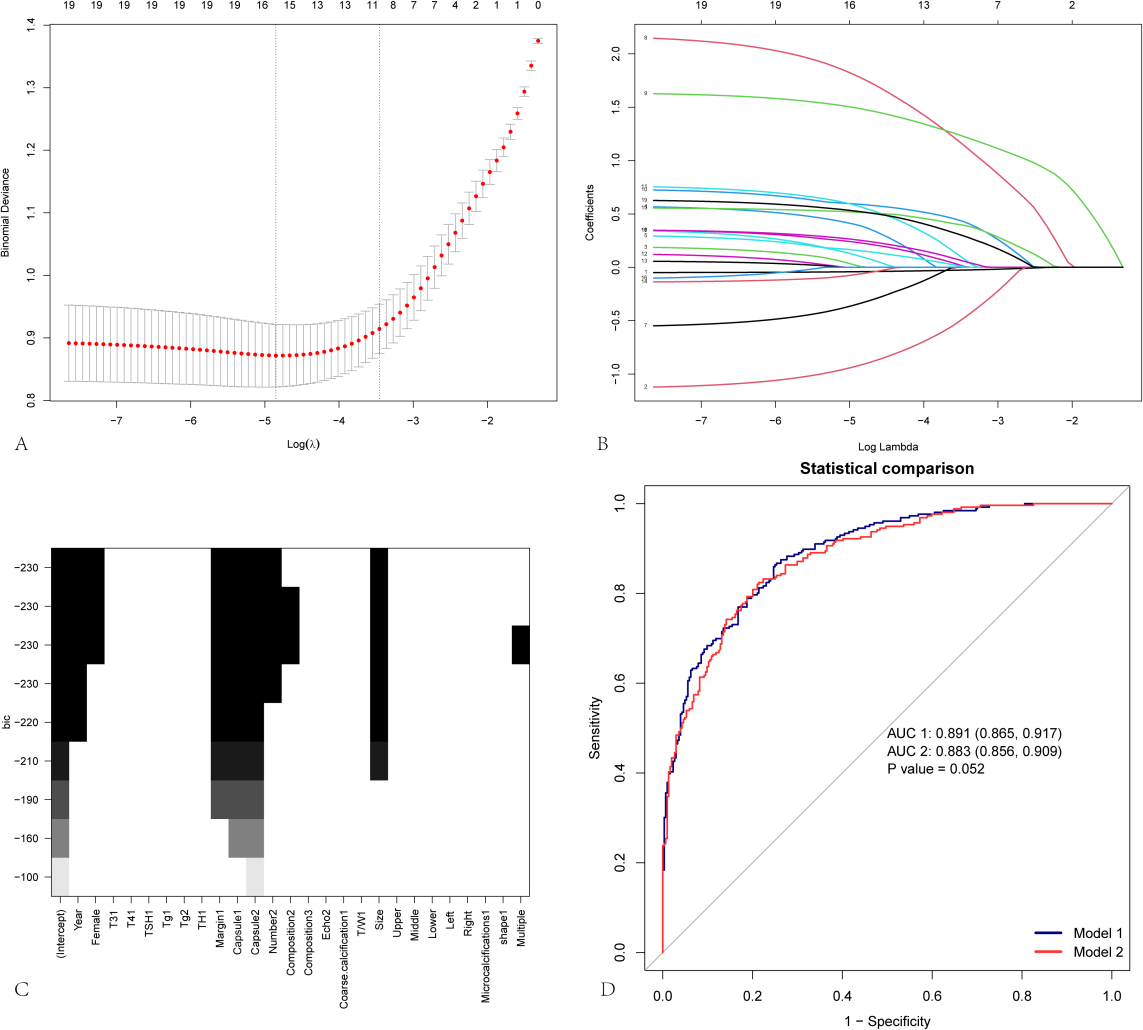
Variable screening for predicting cervical lymph node metastasis. LASSO regression and best subset regression analysis were used to select characteristic factors. **(A)** By verifying the optimal parameter (lambda) in the Lasso model, eleven variables with nonzero coefficients were selected. The mean-square error curve was plotted versus log(lambda), and dotted vertical lines were drawn on the basis of the 1 standard error criterion using the optimal lambda. Lasso refers to the least absolute shrinkage and selection operator. **(B)** A coefficient profile plot was produced for the log(lambda) sequence. **(C)** Best subset selection on the basis of the Bayesian information criterion (BIC). **(D)** Comparison of the ROC curves and AUC values between Model 1 and Model 2. Model 1 is constructed on the basis of 11 variables selected via LASSO regression, whereas Model 2 is based on 7 variables selected via best subset regression (BSR). The AUC values for the two models are 0.891 and 0.883, respectively, with a P value of 0.052, indicating that the difference between the two models is not statistically significant.

We included 560 patients aged 45.5 ± 11.5 years; 77.9% were female, and 22.1% were male. Among all the multifocal nodules, the one with the most characteristic ultrasonography characteristics was chosen for assessment. The overall prevalence of CLNM disease was 45.7%. The baseline demographic clinical data and ultrasound features of the individuals are shown in **Table 1**. Sex, age, Tg, and whole ultrasound characteristics were significantly different between the two groups. We first selected optimal predictors via LASSO regression. When lambda was optimized at 0.032 (1 standard error above the minimum criterion), eleven variables were retained, including age, sex, composition, shape, size, margin, microcalcifications, number, location (left–right distribution), Tg, and contact with the capsule. We then used the BSR to identify potential prognostic factors. The selection of variables was based on the Bayesian information criterion (BIC). Composition, microcalcifications, location (left–right distribution), shape, and Tg were not screened out, and the other factors were approximately consistent with the LASSO regression. We assessed the AUCs for both the six-variable model (0.883, 95% CI: 0.856 to 0.909) and the eleven-variable model (0.891, 95% CI: 0.865 to 0.917), with a P value of 0.052 (**Figure 1**). In line with logistic regression model principles, a good model fit is typically achieved with a smaller set of variables; consequently, we selected six variables as the optimal predictors and incorporated them into our logistic regression model. These factors were ultimately validated as risk factors for CLNM. The kappa coefficient for the ultrasound feature assessment of the thyroid nodules was 0.75.

**Table 1:** Baseline characteristics of the training set and validation set.

Variables	Training set		Validation set	
	- (n = 211)	+ (n = 181)	- (n = 93)	+ (n = 75)
**Age (years)**	47.6 ± 11.0	43.1 ± 11.5	48.7 ± 11.5	41.5 ± 11.1
Sex				
Male	43 (20.4)	52 (28.7)	5 (5.4)	24 (32)
Female	168 (79.6)	129 (71.3)	88 (94.6)	51 (68)
FT3				
Normal	202 (95.7)	169 (93.4)	88 (94.6)	69 (92)
Abnormal	9 (4.3)	12 (6.6)	5 (5.4)	6 (8)
FT4				
Normal	198 (93.8)	168 (92.8)	88 (94.6)	67 (89.3)
Abnormal	13 (6.2)	13 (7.2)	5 (5.4)	8 (10.7)
TSH				
Normal	164 (77.7)	136 (75.1)	71 (76.3)	58 (77.3)
Abnormal	47 (22.3)	45 (24.9)	22 (23.7)	17 (22.7)
Tg				
Normal	145 (68.7)	102 (56.4)	58 (62.4)	49 (65.3)
Mildly elevated	43 (20.4)	38 (21)	20 (21.5)	11 (14.7)
Markedly elevated	23 (10.9)	41 (22.7)	15 (16.1)	15 (20)
HT				
Negative	143 (67.8)	133 (73.5)	65 (69.9)	55 (73.3)
Positive	68 (32.2)	48 (26.5)	28 (30.1)	20 (26.7)
CLNM				
Negative	211 (100)	0 (0)	93 (100)	0 (0)
Positive	0 (0)	181 (100)	0 (0)	75 (100)
Margin				
Smooth	51 (24.2)	5 (2.8)	32 (34.4)	3 (4)
Irregular or ill-definded	160 (75.8)	176 (97.2)	61 (65.6)	72 (96)
Capsule				
Far away	104 (49.3)	14 (7.7)	49 (52.7)	7 (9.3)
Contact with	89 (42.2)	73 (40.3)	34 (36.6)	34 (45.3)
Breakthrough	18 (8.5)	94 (51.9)	10 (10.8)	34 (45.3)
Number				
Single	154 (73)	89 (49.2)	65 (69.9)	39 (52)
Multiple	57 (27)	92 (50.8)	28 (30.1)	36 (48)
Composition				
Solid nodules	202 (95.7)	161 (89)	87 (93.5)	67 (89.3)
Predominantly solid	9 (4.3)	15 (8.3)	5 (5.4)	7 (9.3)
Predominantly cystic	0 (0)	5 (2.8)	1 (1.1)	1 (1.3)
Echogenicity				
Hypoechogenicity	195 (92.4)	153 (84.5)	87 (93.5)	61 (81.3)
Hyperechogenicity	16 (7.6)	28 (15.5)	6 (6.5)	14 (18.7)
Macrocalcification				
Negative	171 (81)	122 (67.4)	70 (75.3)	58 (77.3)
Positive	40 (19)	59 (32.6)	23 (24.7)	17 (22.7)
Microcalcifications				
Negative	72 (34.1)	28 (15.5)	31 (33.3)	11 (14.7)
Positive	139 (65.9)	153 (84.5)	62 (66.7)	64 (85.3)
T/W				
<1	42 (19.9)	55 (30.4)	23 (24.7)	28 (37.3)
≥1	169 (80.1)	126 (69.6)	70 (75.3)	47 (62.7)
**Size (cm)**	0.7 (0.6, 1.0)	1.0 (0.7, 1.7)	0.9 ± 0.4	1.3 ± 0.8
Location 1				
Isthmus	9 (4.3)	6 (3.3)	2 (2.2)	5 (6.7)
Upper	36 (17.1)	32 (17.7)	17 (18.3)	7 (9.3)
Middle	88 (41.7)	69 (38.1)	38 (40.9)	29 (38.7)
Lower	49 (23.2)	24 (13.3)	25 (26.9)	17 (22.7)
Multifocal lesions	29 (13.7)	50 (27.6)	11 (11.8)	17 (22.7)
Location 2				
Isthmus	9 (4.3)	6 (3.3)	2 (2.2)	5 (6.7)
Left lobe	72 (34.1)	57 (31.5)	44 (47.3)	20 (26.7)
Right lobe	104 (49.3)	73 (40.3)	39 (41.9)	36 (48)
Multifocal lesions	26 (12.3)	45 (24.9)	8 (8.6)	14 (18.7)

FT4, free thyroxine; FT3, free triiodothyronine; TSH, thyroid stimulating hormone; Tg, thyroglobulin; HT, Hashimoto's thyroiditis; CLNM, cervical central lymph node metastasis; T/W, taller than wide.

Logistic regression analysis of these factors (**Tables 2**, **3**) revealed that women had an odds ratio (OR) of 0.3 (0.17–0.53) for CLNM compared with men. The OR for CLNM was 12.2 for ill-defined margins compared with smooth margins. The OR for CLNM was 5.42 for tumors in contact with the capsule and 28.37 for tumor invasion and invasion or breakthrough of the capsule compared with tumors located far from the capsule. Moreover, the incidence of CLNM increased by 5% with decreasing age. There was also a 119% increase in the incidence of multifocal lesions and a 245% increase in the incidence when the nodule diameter increased by one centimeter.

**Table 2:** Multivariate logistic regression analysis of risk factors associated with CLNM.

Variable	crude.OR (95%CI)	crude.P value	adj.OR (95%CI)	adj.P value
**Age**	0.96 (0.94~0.97)	<0.001	0.95 (0.93~0.97)	<0.001
Sex				
Male	1 (Ref)		1 (Ref)	
Female	0.44 (0.3~0.67)	<0.001	0.3 (0.17~0.53)	<0.001
Margin				
Smooth	1 (Ref)		1 (Ref)	
Irregular	11.64 (5.51~24.59)	<0.001	12.2 (4.84~30.77)	<0.001
Capsule				
Far away	1 (Ref)		1 (Ref)	
Contact with	6.34 (3.75~10.71)	<0.001	5.42 (3.05~9.63)	<0.001
Breakthrough	33.31 (18.05~61.46)	<0.001	28.37 (14.19~56.7)	<0.001
Number				
Single	1 (Ref)		1 (Ref)	
Multiple	2.58 (1.81~3.66)	<0.001	2.19 (1.35~3.55)	0.002
**Size (cm)**	3.34 (2.38~4.68)	<0.001	3.45 (2.23~5.31)	<0.001

Crude model: no other covariates were adjusted.

Adjusted model: we adjusted age, sex, and all thyroid serological indicators.

**Table 3:** Logistic analyses of the risk factors and CLNM.

Item	Estimate	Std.Error	Z.value	P.value
(Intercept)	-2.251	0.869	-2.591	0.01
Size	0.835	0.247	3.385	0.001
Age	-0.054	0.013	-4.278	0
Sex	-0.872	0.324	-2.693	0.007
Ill-defined margin	2.537	0.615	4.121	0
Capsule				
Contact with	1.64	0.365	4.493	0
Breakthrough	3.198	0.433	7.387	0
Multifocal lesions	1.013	0.287	3.534	0

**Figure 2:**
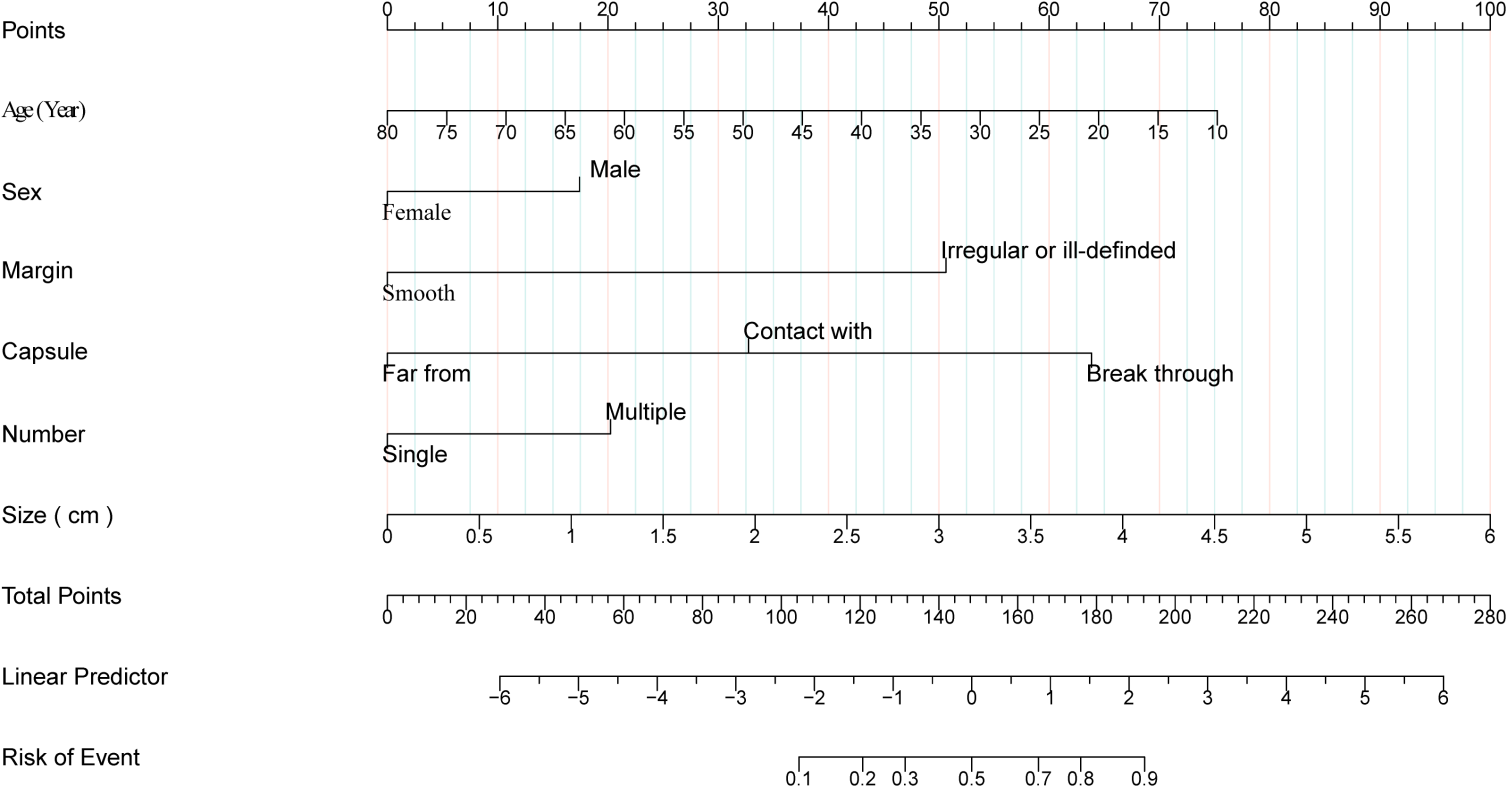
Nomogram of cervical lymph node metastases in papillary thyroid carcinoma. Clinical and ultrasound factor-based nomograms used for preoperatively predicting CLNM in PTC patients.

The nomogram was created via six prognostic factors derived from multivariate logistic regression analysis (**Figure 2**). Summing the individual scores for each variable yields a cumulative total, which corresponds to a specific value on the risk axis indicative of the likelihood of CLNM in patients with PTC. An elevated score signifies an increased risk of CLNM for PTC patients. For example, the following results were obtained: male, 35 years old, largest diameter=1.5 cm, multifocal lesions, contact with the capsule, and ill-defined margins. The corresponding risk for CLNM was 73% (**Supplementary Figure 1**).

**Figure 3:**
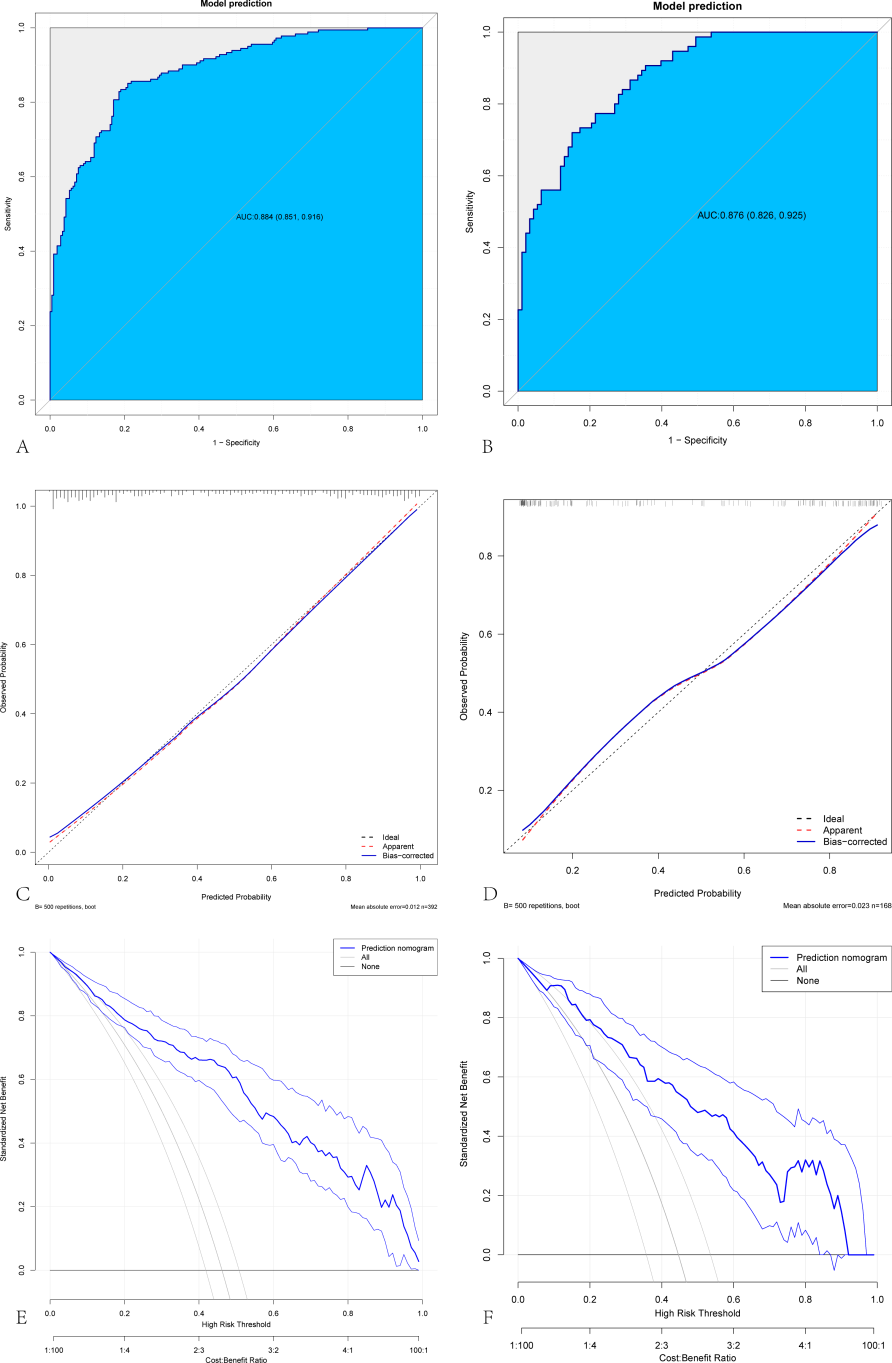
Internal validation of a predictive model for cervical lymph node metastasis in papillary thyroid carcinoma. Evaluation of the prediction model on the training set and internal validation set. **(A, B)** ROC curves of the CLNM prediction model in the training and internal cross-validation sets. **(C, D)** Calibration curves of CLNM for papillary thyroid cancer in the training and internal cross-validation sets. **(E, F)** The decision curve of CLNM for papillary thyroid cancer in the training and internal cross-validation sets.

We performed 7:3 random split verification and bootstrapping to evaluate the model. The nomogram was assessed via the area under the curve (AUC) and Brier value. The Briers for the training set, internal cross-validation test set and external validation test set were 0.138, 0.134, and 0.103, respectively (**Table 4**). To assess the accuracy of the model, **Figure 3** shows the ROC curves, clinical decision curves, and calibration curves for the training and test groups. The AUC of the model was 0.884 (0.851, 0.916) in the training set and 0.876 (0.826, 0.925) in the testing set (**Figures 3A, B**). The model’s calibration demonstrated satisfactory agreement, as evidenced by the nonsignificant Hosmer–Lemeshow test results for both the training set (*χ^2^* = 6.791, *p* = 0.559) and testing set (*χ^2^* = 11.179, *p* = 0.692) (**Figures 3C, D**; **Table 4**). A decision curve revealed that patients received a net clinical benefit (**Figures 3E, F**). **Supplementary Figure 2** shows the AUCs of the training set and the internal and external test sets. Overall, the plots indicate that all three datasets exhibit strong performance across discrimination, calibration, and decision-making utility.

**Table 4:** Predictive performance of the final prediction model on the basis of random split and bootstrap verification.

Model Performance	ROC (95% CI)	Brier	Hoslem Test X-squared	Hoslem Test P value
Original performance	0.884 (0.851, 0.916)	0.138	6.791	0.559
Internal cross validation	0.876 (0.826, 0.925)	0.134	11.179	0.192
External Validation	0.899 (0.844,0.954)	0.103	5.067	0.75

## Discussion

Although the prognosis of PTC is relatively good, CLNM often occurs in the early stage of the disease (8). Identifying independent risk factors for CLNM is therefore critical, as it can help guide clinical management and treatment decisions for thyroid nodules. In this study, we constructed and validated a nomogram using factors such as age, sex, tumor size, margin, number of tumors and whether the tumor was in contact with or had invaded the capsule. To predict CLNM, this nomogram was assessed via the AUC, decision curve and calibration curve. Our research demonstrated that the nomogram has strong discriminative power, satisfactory calibration performance, and practical clinical applicability.

Investigators have developed predictive models based on laboratory biochemical indicators or ultrasound features to help clinicians identify patients with a greater likelihood of CLNM (9, 10). Hu et al. reported that the levels of FT3, TPOAb and Tg were significantly different between patients with and without LNM, and multifactorial regression analysis revealed that Tg was an independent predictor of LNM (10). In our study, no significant difference in Tg levels was observed between the metastatic and nonmetastatic groups, indicating that Tg is not an independent predictor; therefore, the authors suggest that whether serum Tg can be used as a routine preoperative measurement to assess the aggressiveness of thyroid nodules needs to be confirmed by further studies. Previous studies have reported conflicting findings regarding the correlation between HT and CLNM in patients with PTC (11–13). Some researchers have reported a positive association, suggesting that the autoimmune response to HT, which elevates TSH levels, may promote PTC progression and invasion as TSH levels increase (14–16). Other studies have concluded that patients with HT are at a significantly greater risk of developing PTC and that the disease tends to be more indolent (17, 18). HT was not identified as a significant predictor of CLNM in our study. Consequently, the nomogram reflects the absence of HT as a contributing factor to the prediction of CLNM.

More research has been conducted on the usefulness of PTC ultrasonography characteristics in predicting CLNM (19–23). Zhou et al. reported that maximum tumor diameter, microcalcification and irregular margins were independent risk factors for CLNM (24). The average maximum diameters of the tumors in the positive and negative groups were 13.3 mm and 8.5 mm, respectively. In contrast, our study found these values to be 10.0 mm and 7.0 mm, with a cutoff value of 7.5 mm (**Table 5**). The possible reason is that most patients who underwent surgery had thyroid nodules greater than 1.0 cm in their research but less than 1.0 cm in our study. The definition of the nodule size cutoff value (1.0 cm) aims to avoid excessive diagnosis and overtreatment of low-risk indolent PTC, but it also risks neglecting early-stage invasive cancers (25). However, this cutoff value has not been widely accepted (26, 27). In light of this, clinicians should be vigilant in treating CLNM when the nodule diameter is greater than 0.75 cm to avoid inadequate treatment. The histological features of the tumor margin reflect the invasiveness of the tumor (28). This feature is particularly evident in thyroid cancer. Our study results also confirmed this, and the underlying mechanism may be that malignant tumor cells grow rapidly and unevenly, leading to an irregular shape and unclear margins of the nodule.

**Table 5:** The predictive value of age and nodule size for CLNM.

Variable	n	AUC	Specificity	Sensitivity	Youden	Threshold
Age	560	0.632	0.655	0.590	1.244	44.5
Size	560	0.691	0.576	0.719	1.294	0.745

Previous studies have been controversial regarding whether the location of the nodule is associated with CLNM (22, 29, 30). Our study revealed that the location of the nodule is not a significant predictor of CLNM, which is consistent with the findings of Chen’s study (31). The number of lesions is associated with the invasive characteristics of the nodules. Patients with multiple foci have a greater risk of lymph node metastasis, vascular invasion, and extrathyroidal extension (32, 33). Our study results further support the viewpoint that multifocality serves as a significant predictor of CLNM. Additionally, microcalcifications, coarse calcifications, echo, composition, and T/W were significantly different between the lymph node metastasis set and the nonmetastasis set, but multifactorial regression analysis revealed that these features were not independent risk factors for CLNM.

The adjacent capsule, especially the breakthrough capsule, can predict CLNM, especially in male patients (4, 23, 34, 35). This is because thyroid cancer tissues can express matrix metalloproteinases (MMPs), which disrupt the extracellular matrix and basement membrane components, facilitating invasion into the surrounding stroma (36, 37). The encroachment of a nodule upon the thyroid capsule results in a breach of its continuous structure. Consequently, the nodule may eventually penetrate the capsule, facilitating its spread to adjacent tissues and regional lymph nodes (38). With respect to the impact of sex hormones on PTC, bioinformatics and fundamental experimental results suggest that testosterone secretion promotes the growth and invasive malignant behavior of PTC (39); therefore, being male may imply a greater likelihood of metastasis (40). In PTC, age stands out as a highly significant prognostic factor, with younger patients being more likely to experience lateral neck lymph node metastasis (41). Genetic factors may also contribute to the varying incidence rates of lymph node metastasis (LNM) in papillary thyroid carcinoma (PTC) across different ages and sexes. For example, certain populations might exhibit a higher prevalence of specific genetic mutations or elevated expression of particular channel proteins. These genetic and protein profiles can interact with age and sex, thereby influencing the progression of the disease (42, 43). Most studies employ 45 years or 55 years as the cutoff for staging. This method is based on the American Joint Committee on Cancer/Tumor-Node-Metastasis (AJCC/TNM; www.cancerstaging.org) system, which is the most widely used method for predicting disease-specific survival in patients with differentiated thyroid cancer. When age is considered as a continuous variable, the cutoff values obtained from different studies also tend to be close to these two values (8, 44), which is consistent with our study results. Consequently, it is essential for ultrasonographers to meticulously assess the interface between nodules and the thyroid capsule especially in young males. Such a detailed examination enhances the precision of prognostic information pertaining to cervical lymph node metastasis (CLNM), which is conveyed to surgical teams (38, 45).

Clinical laboratory and ultrasound indicators were jointly predicted in our investigation. This approach overcomes the limitations of single predictors in previous research (8, 46, 47). While these models have shown moderate success, they may not capture the full complexity of the disease dynamics. Additionally, the prediction model demonstrated excellent generalizability and clinical applicability in preliminary validation. By predicting the risk of lymph node metastasis preoperatively, our model can guide clinicians in determining whether to perform lymph node dissection. This precise risk assessment helps avoid over-treatment or under-treatment, ensuring that patients receive the most appropriate care.

Nevertheless, several limitations should be acknowledged. First, its retrospective nature makes it susceptible to selection bias. For example, potential confounding factors such as environmental pollution and genetic mutations were not considered in this study, which may have influenced our results. Second, while we expanded our dataset by incorporating cases from another medical institution for external validation, the total number of PTC patients included in this study was relatively limited. Although this addition represents a step forward, our data are still limited to only two centers. To ensure the robust clinical application of our model, it is essential to further expand the dataset to include cases from multiple centers. This will provide a more comprehensive and diverse patient population, thereby enhancing the generalizability and reliability of our findings. In addition, although the nomogram and decision curve analysis in this study demonstrated the substantial clinical utility of the model, enabling accurate preoperative prediction of CLNM through clinical assessment and thus achieving clinical benefits, challenges related to standardization still persist. Therefore, comprehensively strengthening standardized operating procedures and applying validated and widely recognized thyroid imaging data systems to promote consistency in data evaluation are recommended.

## Conclusions

A prognostic tool and nomogram for estimating CLNM in patients with PTC were developed and demonstrated robust performance in terms of discrimination and calibration, achieving very high diagnostic efficacy in the internal and external validation groups and providing a basis to help clinicians better manage PTC patients.

## Data Availability

The raw data supporting the conclusions of this article will be made available by the authors, without undue reservation.

## Supplementary material

Supplementary Figure 1 Clinical usage of our nomogram.

Supplementary Figure 2 Model performance evaluation across different datasets: ROC curves and calibration curves **(A-C)** ROC curves for the model evaluated on the training dataset, internal validation dataset, and external validation dataset. **(D-F)** Calibration curves for the training set, internal validation set, and external validation set, respectively.
