# Supplementary figures and images for: Construction of a predictive model for cervical lymph node metastasis in papillary thyroid carcinoma
Source: Front Oncol. 2025 May 15;15:1549148. doi: 10.3389/fonc.2025.1549148 (PMC12119560; doi:10.3389/fonc.2025.1549148)

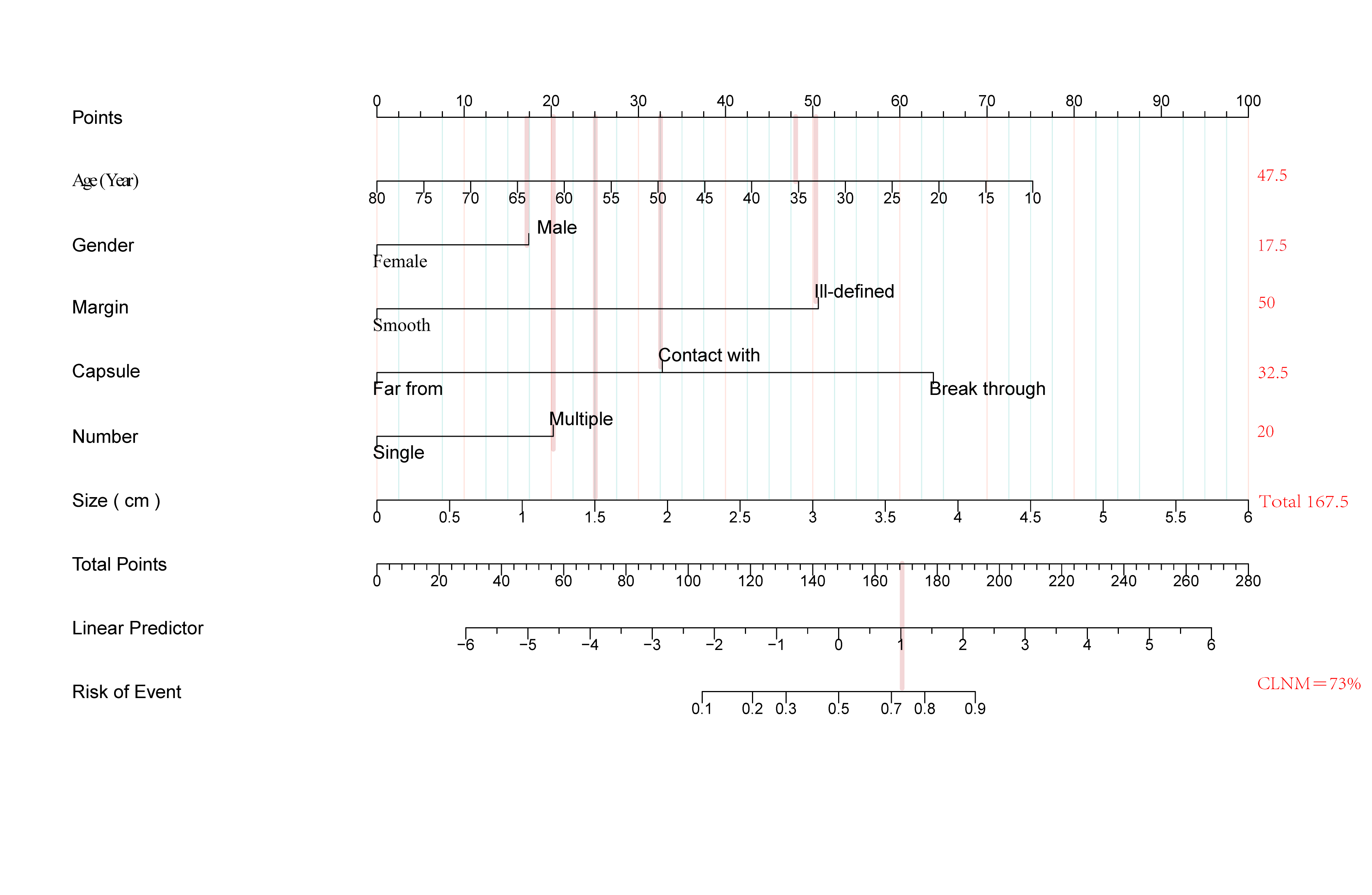

Supplement: Supplementary Figure 1 — Clinical usage of our nomogram. [file Image1.tif]

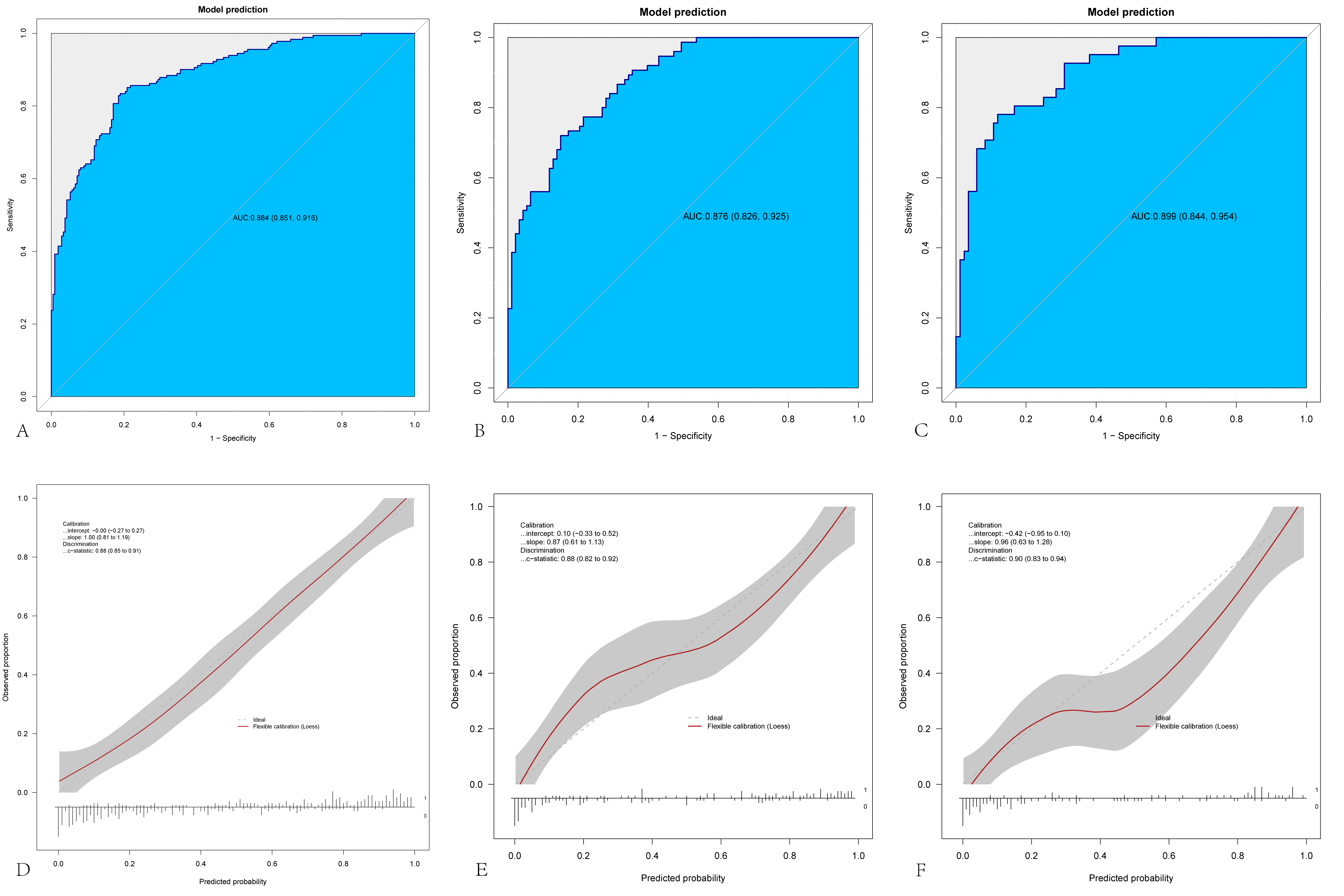

Supplement: Supplementary Figure 2 — Model performance evaluation across different datasets: ROC curves and calibration curves (A-C) ROC curves for the model evaluated on the training dataset, internal validation dataset, and external validation dataset. (D-F) Calibration curves for the training set, internal validation set, and external validation set, respectively. [file Image2.tif]
